# The antifungal activity and mechanism of silver nanoparticles against four pathogens causing kiwifruit post-harvest rot

**DOI:** 10.3389/fmicb.2022.988633

**Published:** 2022-08-31

**Authors:** Li Li, Hui Pan, Lei Deng, Guoliang Qian, Zupeng Wang, Wenyi Li, Caihong Zhong

**Affiliations:** ^1^Key Laboratory of Plant Germplasm Enhancement and Specialty Agriculture, Engineering Laboratory for Kiwifruit Industrial Technology, Wuhan Botanical Garden, Chinese Academy of Sciences, Wuhan, China; ^2^College of Plant Protection, Nanjing Agricultural University, Nanjing, China

**Keywords:** nanoparticles, kiwifruit post-harvest rot, antifungal activity, transcriptomic analysis, microstructure observation

## Abstract

Post-harvest rot causes enormous economic loss to the global kiwifruit industry. Currently, there are no effective fungicides to combat the disease. It is unclear whether silver nanoparticles (AgNPs) are effective in controlling post-harvest rot and, if so, what the underlying antifungal mechanism is. Our results indicated that 75 ppm AgNPs effectively inhibited the mycelial growth and spore germination of four kiwifruit rot pathogens: *Alternaria alternata*, *Pestalotiopsis microspora*, *Diaporthe actinidiae*, and *Botryosphaeria dothidea*. Additionally, AgNPs increased the permeability of mycelium’s cell membrane, indicating the leakage of intracellular substance. Scanning electron microscopy (SEM) and transmission electron microscopy (TEM) observations revealed that AgNPs induced pathogen hypha shrinkage and distortion, as well as vacuolation in hypha cells, implying that AgNPs caused cellular and organelle structural degradation. The transcriptome sequencing of mycelium treated with AgNPs (24 h / 48 h) was performed on the Illumina Hiseq 4000 sequencing (RNA-Seq) platform. For the time points of 24 h and 48 h, AgNPs treatment resulted in 1,178 and 1,461 differentially expressed genes (DEGs) of *A. alternata*, 517 and 91 DEGs of *P. microspora*, 1,287 and 65 DEGs of *D. actinidiae*, 239 and 55 DEGs of *B. dothidea*, respectively. The DEGs were found to be involved in “catalytic activity,” “small molecule binding,” “metal ion binding,” “transporter activity,” “cellular component organization,” “protein metabolic process,” “carbohydrate metabolic process,” and “establishment of localization.” Kyoto Encyclopedia of Genes and Genomes (KEGG) analysis also revealed that “carbohydrate metabolism,” “amino acid metabolism,” “energy metabolism,” and “xenobiotics biodegradation and metabolism” of “metabolism processes” were the most highly enriched pathways for these DEGs in four pathogens, with “cellular processes” being particularly enriched for *B. dothidea.* Furthermore, quantitative polymerase chain reactions (qPCRs) were used to validate the RNA-seq results. It was also confirmed that AgNPs could significantly reduce the symptoms of kiwifruit rot without leaving any Ag^+^ residue on the peel and flesh of kiwifruit. Our findings contributed to a better understanding of the antifungal effect and molecular mechanisms of AgNPs against pathogens causing kiwifruit post-harvest rot, as well as a new perspective on the application of this novel antifungal alternative to fruit disease control.

## Introduction

Kiwifruit is popular with consumers due to its unique flavor and high concentration of vitamin C, minerals, and other nutrients (Diaz et al., 2017). By 2021, the global kiwifruit planting area, yield, and output value have reached 270,500 hectares, 4407,400 tons, and USD 10 billion, respectively. However, as demand grows and the kiwifruit producing area in China expands, post-harvest rot diseases become more severe, with the average infected rate reaching 30–50%, and causing more than 100,000 tons of fruit losses per year, severely limiting the industry’s healthy development (Li et al., 2017a; Dai et al., 2021).

Several fungi have been identified as pathogens of kiwifruit rot disease, including *Botryosphaeria dothidea* (Zhou et al., 2015; Pan et al., 2020), *Alternaria alternata* (Li et al., 2017b; Gao et al., 2021), *Pestalotiopsis microspora* (Li et al., 2016) and *Diaporthe* sp. (*D. ambigua*, *D. australafricana*, *D. novem*, *D. rudis* and *D. actinidiae*) (Diaz et al., 2017; Li et al., 2017c). In recent years, the pathogens are generally controlled by the pre-harvest application of chemical fungicides such as Tebuconazole and Carbendazim (Kim et al., 2013). However, overuse of such fungicides has resulted in a significant increase in pathogen resistance to these fungicides, as well as fungicide residues on fruits, both of which are detrimental to public health and the environment (Chen et al., 2020; Kai et al., 2020). Therefore, it is critical to develop an effective and safe alternative to control kiwifruit post-harvest rot.

Because of the high efficiency, broad-spectrum antimicrobial activity, minimal resistance, and excellent safety, silver nanoparticles (AgNPs) have been widely used for plant disease control in recent years (Tian et al., 2018; Gordienko et al., 2019; Al-khattaf, 2021). AgNPs inhibited the growth of plant pathogenic fungi including *Fragaria ananassa* (Moussa et al., 2013), *Pyricularia* sp. (Lee et al., 2013), *Aspergillus niger* (Xue et al., 2016), *Botrytis cinerea* (Malandrakis et al., 2019), and *Fusarium* sp. (Shen et al., 2020; Jian et al., 2021). AgNPs’ antifungal mechanism is primarily attributed to inhibiting mycelial growth and conidial germination, degrading cell walls and membranes, disputing protein, producing reactive oxygen species (ROS), influencing pathogen energy and substance metabolism, signal transduction, and genetic information processing (Jian et al., 2021; Al-Otibi et al., 2022). However, the mechanism of AgNPs against plant pathogens remains unknown, owing to a lack of microscopic, biochemical, and omic analysis (Shen et al., 2020).

In recent years, AgNPs have been applied to control post-harvest diseases in citrus (Al-Sheikh and Yehia, 2016), mango (Shivamogga Nagaraju et al., 2020), apple (Madbouly, 2021), and strawberry (Oliveira et al., 2022). However, no AgNPs research on preventing kiwifruit rot has been published. *B. dothidea, D. actinidiae, P. microspora* and *A. alternata* have been identified as pathogens causing kiwifruit post-harvest rot in our preliminary study (Li et al., 2017c). Previous research found that AgNPs could directly inhibit mycelial growth and spore germination of *A.alternata*, as well as affect cell wall composition and the synthesis of sugar, protein, and N-acetylglucosamine (Ouda, 2014; Ganash et al., 2018). 150 ppm AgNPs (mean size 10 ± 5 nm) had potent antifungal activity against *A.alternata* when compared to Iprodione and Difenoconazole; 180 ppm AgNPs (mean size 52 nm) inhibited the growth of *Diaporthe* sp. (Mendes et al., 2014; Abdelmalek and Salaheldin, 2016). However, there have been no systematic studies on the effect of AgNPs on pathogens causing kiwifruit post-harvest rot, especially *B. dothidea*, *D. actinidiae*, and *P. microspora*.

In this study, we first determined the antifungal capacity of AgNPs against four pathogens causing kiwifruit post-harvest rot. Then, using an electron microscope and transcriptome profiling, we identified the cytological and molecular mechanisms by which AgNPs suppressed the above pathogens. Finally, we investigated the antifungal efficacy and safety of AgNPs applied to kiwifruit. The study verified that AgNPs can be used to control kiwifruit rot disease, and laid the groundwork for the application of AgNPs to control other fruit fungal diseases.

## Materials and methods

### Silver nanoparticles suspension

The polyvinyl pyrrolidone (PVP, 40KDa) coating AgNPs stock (5 mg mL^–1^) purchased from NanoComposix (San Diego, CA, United States) had an average diameter of 5 nm and was stored at 4°C, away from light (Lu et al., 2022). Different concentrations of AgNPs solutions were prepared with sterile distilled water (SDW).

### Pathogenic fungi

Four strains isolated from infected fruits were identified as pathogens causing kiwifruit post-harvest rot by analyzing morphological characteristics and nucleotide sequences containing the internal transcribed spacer rDNA region (ITS), β-tubulin, and elongation factor-1 alpha gene (EF-1α), including *B. dothidea, D. actinidiae, P. microspora* and *A. alternata* (Li et al., 2016, 2017a,2017b,2017c). Four strains were cultured on potato dextrose agar (PDA, extract of potatoes 200 g, dextrose 20 g, agar 20 g per liter) for 14 days at 25°. The spore suspensions were made by rinsing the spores and hypha in 10 mL SDW and filtering them through two layers of sterile gauze to remove any adherent mycelium. The concentration of the homogenous spore suspension was measured by a hemocytometer and diluted to 1.0 × 10^6^ spores mL^–1^.

### Kiwifruit

Yellow fleshed fruits of *Actinidia chinensis* cv ‘Jinmei’ were collected from the scientific research orchard of Engineering Laboratory for Kiwifruit Industrial Technology, Chinese Academy of Sciences (CAS). Fruits with a consistent shape, size, ripeness, and lack of visible mechanical wounds were selected.

### Antifungal effect of silver nanoparticles on mycelial growth of pathogens

The antifungal activity of AgNPs was investigated by measuring the diameter of fungal colonies (Wollenberg et al., 2016). The 7 mm diameter mycelial block was taken from a 1-week-old culture and placed in the center of a PDA petri dish (diameter = 90 mm) containing 25 mL of PDA with various AgNPs concentrations (0, 0.5, 5, 15, 30, 45, 60, 75, 90 ppm). Cultures were incubated at 25° and colony diameter was measured using the cross method on the sixth day after inoculation. The PDA plate without AgNPs was used as the control. Each treatment performed with three replicates, and the experiment was repeated twice. The inhibition rate of mycelium growth was calculated as follows (Alothman and Abd-Ei-Aziz Abeer, 2019):


Inhibitionrate(%)=

$$\frac{\left(\mathrm{control}\,\mathrm{colony}\,\mathrm{diameter}-\mathrm{treated}\,\mathrm{colony}\,\mathrm{diameter}\right)}{\mathrm{control}\,\mathrm{colony}\,\mathrm{diameter}}\times 100$$

EC_50_ (effective concentration of 50% inhibition rate) values were calculated statistically using SPSS Statistics 21 (SPSS Inc., United States).

### Antifungal effect of silver nanoparticles on spore germination and germ tube elongation

The inhibition of AgNPs on spore germination was observed with modification of He et al. (2019). According to the analysis result of 2.4, the optimal inhibitory concentration of AgNPs for four strains was 75 ppm. For each pathogen, a 100 μL spore suspension (1.0 × 10^6^ spores mL^–1^) was prepared and inoculated into tubes containing 1 mL potato dextrose broth (PDB, extract of potatoes 200 g and dextrose 20 g per liter) containing 75 ppm AgNPs, with the final concentration adjusted to 1.0 × 10^5^ spores mL^–1^. PDB without AgNPs served as the negative control. The tubes were then cultured in a constant temperature shaker at 25°C at a speed of 180 r/min. The spore germination and germ tube elongation were then observed using an optical microscope (OLYMPUS BX51, Olympus Corporation, Japan).

As the spore germination period of different pathogens varies, the observation time was also modified based on the growth curve and set as follows: *B. dothidea* 2 h, *D. actinidiae* 4 h, *P. microspora* 5 h, and *A. alternata* 9 h. If the germ tube length is equal to or longer than the spore length, germination is considered. The percentage in germination was observed with modification of Al-Sheikh and Yehia (2016). The test was repeated twice with three replicates each, with approximately 100 spores constituting one replicate.

### Antifungal effect of silver nanoparticles on membrane permeability of mycelium

Mycelium permeability was measured using methods described by Zhang et al. (2007) with little modification. For each strain, 0.4 g mycelium was mixed in 15 mL of 75 ppm AgNPs in a tube before being placed in a thermostatic bath at 26°. Electrical conductivity was measured at 0, 1, 3, 6, 12, 24, and 48 h, finally after boiling. Mycelium in SDW served as a control. Each three-parallel treatment was repeated three times.


Relativepermeability(%)=

$$\frac{\mathrm{Treatment}\,\mathrm{conductivity}\,\mathrm{Initial}\,\mathrm{conductivity}}{\mathrm{Boiling}\,\mathrm{conductivity}\,\mathrm{Initial}\,\mathrm{conductivity}}\times 100$$

### Antifungal effect of silver nanoparticles on mycelial morphology and ultrastructure

Scanning Electron Microscope (SEM) and Transmission Electron Microscope (TEM) were used to examine the effect of AgNPs on the morphological and ultrastructural alterations of four pathogens, as described before (Li et al., 2021). The strains were cultured on PDA plates with 75 ppm AgNPs for 3 d at 25°. PDA dishes without AgNPs were used as control. Mycelial fragments (approximately 3∼5 mm) were collected from the colony’s edge and fixed in 2.5 % glutaraldehyde at 4° for 12 h, then rinsed three times with PBS (pH 7.2, 0.01M) for further processing.

For SEM analysis, mycelia samples from each treatment were dehydrated in an ethanol series (30, 50, 70, and 90%, v/v) for 20 min at each phase, followed by centrifugation. Following drying at the critical point, samples were attached to the sample stage with conductive glue. Lastly, specimens were sputtered with palladium-gold to observe under SEM (JEOL JSM-6390LV, Japan).

For TEM investigation, mycelia samples were postfixed with 1% osmic acid at 4° for 3 h, rinsed three times with PBS, and dehydrated with graded acetone solutions (30, 50, 70, 80, and 90%, v/v), each stage lasting 30 min. The specimens were penetrated for 12 h at each stage with a volume gradient of acetone and embedding medium (5:1, 3:1, 1:1, 1:3, 1:5, v/v), followed by pure embedding medium. Then, the specimens were embedded in SPI-Pon 812, ultrasliced using an ultramicrotome (Leica EM UC6, Germany), and stained with uranyl acetate to detect under TEM (Hitachi H-7650, Japan).

### Antifungal effect of silver nanoparticles against pathogens by RNA-seq

The flow of the transcriptomics experiment included extraction of total RNA; enrichment of mRNA by Oligo (dT); RNA fragment; random hexamer primed cDNA synthesis; size selection and PCR amplification; and sequencing on an Illumina HiSeq 4000 (Zhao et al., 2022). The samples were collected, 0.4 g mycelium was inoculated into 15 mL PDB medium containing 75 ppm AgNPs for each strain, and cultured at 25° for 24 h (Ag-24h) and 48 h (Ag-48h). Control mycelium was grown in PDB without AgNPs for 24 h (CK-24h) and 48 h (CK-48h). Thus, 16 treatments with three repetitions were established, yielding 48 samples in total. Total RNA from 48 samples was extracted by using TRIzol (Invitrogen, Carlsbad, CA, United States) according to the manufacturer’s instruction. To eliminate DNA from total RNA samples, Invitrogen TM RNase-free DNase I was utilized. mRNA was extracted from total RNA using poly-T oligo-attached magnetic beads and then fragmented into 200–300 bp fragments to produce strand-specific cDNA libraries using a TruSeq RNA Sample Preparation Kit (Illumina, San Diego, CA, United States). Finally, the cDNA was paired-end sequenced on the Illumina Hiseq 4000 sequencing platform using the PE150 sequencing strategy (Patnaik et al., 2016).

To obtain clean data, the two ends of raw reads with low-quality bases or adapters were truncated by Trimmomtic v0.39 (Bolger et al., 2014) with parameters “ILLUMINACLIP: adapters/TruSeq3-PE-2.fa:2:30:10 LEADING:5 TRAILING:5 SLIDINGWINDOW:4:15 MINLEN:50 TOPHRED33.” The clean data were then aligned to the reference genome sequence using HISAT2 v2.1.0 (Kim et al., 2019) with parameters “–rna-strandness RF –min-intronlen 20 –max-intronlen 4000 –dta –dta-cufflinks –score-min L,0.0, −0.4.” The HISAT2 alignment results were used to obtain raw count expression results by HTseq v0.13.5 software (Anders et al., 2014) with parameters “-f sam -r name -s reverse -a 10 -t exon -i gene_id.” And then, the raw counts matrix data of 48 samples were normalized by two methods sequentially, using the TPM (Transcripts Per Kilobase Million) for eliminating gene length differences and TMM (Trimmed Mean of *M*-values) (Robinson and Oshlack, 2010) for cross-comparison among samples. Finally, the edgeR v3.22.5 (Robinson et al., 2010) and DESeq2 v1.20.0 (Love et al., 2014) software were utilized to compare the raw counts of gene expression and calculate the different significance between samples. The differentially expressed genes (DEGs) (Shen et al., 2020) were identified with false discovery rate (FDR) = 0.01 both in edgeR and DESeq2 methods and absolute log2 fold change of TPM/TMM gene expression=1 (Zhao et al., 2019, 2020). The identified DEGs were then subjected to hierarchical clustering, as well as Gene Ontology (GO)^1^ and Kyoto Encyclopedia of Genes and Genomes (KEGG)^2^ pathway enrichment analyses (Wang et al., 2021).

RT-qPCR was performed to validate the transcriptional level results acquired by RNA sequencing. Total RNA was extracted in the same RNA-Seq samples and reverse-transcribed into cDNA, and then used for RT-qPCR using SYBR Premix Ex Taq II (Ke et al., 2014). The genes with the maximum FDR value and average TPM value between 20 and 40 (the upper quartile of all genes TPM expression was about 30) were selected as reference in RT-qPCR, and eight DEGs with the minimum FDR value and average TPM value between 20 and 40 were chosen for validation (Supplementary Table 1). Additionally, three stable reference genes were used to determine the relative quantification of each target gene (Supplementary Table 1). On a 7500 Real-Time PCR system (ABI, United States), the relative expression of the genes was measured three times, each time with three technical replicates (Yin et al., 2015). The target genes’ expression levels were calculated by the 2^–ΔΔCT^ method (Livak and Schmittgen, 2001).

### Antifungal effect and safety of silver nanoparticles on kiwifruit rot disease

In our previous study, we successfully constructed the fluorescence strain of *P. microspora*, which could be used for the detection of infection process (Deng et al., 2021). *Agrobacterium tumefaciens* mediated transformation of *P. microspora* strain was carried out by using recombinant vector pCAMBgfp containing hygromycin resistance gene and GFP lable. The fluorescent transformant PM-13 has no significant differences in morphological characteristics and pathogenicity as compared with the wild-type strain.

The control efficacy of AgNPs against post-harvest rot in kiwifruit ‘Jinmei’ was assessed as previously recorded (Pan et al., 2020) with modification. Fruits were disinfected in 0.2% sodium hypochlorite solution (v/v) for 2 min, rinsed 5 times with sterilized water, and air-dried at room temperature. Then, fruits were inoculated with 10 μL 1.0 × 10^6^ spores mL^–1^ suspension of *P. microspora* fluorescent strain PM-13. 75 ppm AgNPs were sprayed on fruits 24 h before or after pathogen inoculation, with control fruits not treated with AgNPs. After air-drying, all treated fruits were placed in a plastic box at 22° with a high relative humidity (approximately 80%). On the sixth post-inoculation day, the fruit peel was scraped and the disease lesions were measured before being photographed using a LUYOR-3410 fluorescence excitation source. Each treatment was performed three times, with three fruits per replicate.

As described previously, the Microwave Digestion-ICP-MS method was used to examine the Ag^+^ residue on kiwifruit (Navarro et al., 2006; Aldabe et al., 2013). The AgNPs-treated and control fruits (inner/external fruit flesh and peel) were crushed with liquid nitrogen, dried at 60°, and sieved through a 100-mesh screen. For each treatment, 0.25 g powder was added, respectively into test tubes with 5 mL nitric acid and 1 mL hydrogen peroxide, then digested with a Microwave Sample Preparation System (ETHOS ONE, Milestone) and the acid was exorcized. Subsequently, the solution was diluted with 1% HNO_3_ in a 50 mL volumetric flask, filtered, and the Ag^+^ content of the fruit sample was determined by Inductively Coupled Plasma Mass Spectrometry (ICP-MS, Thermo Fisher Scientific, X Series 2). The national hygienic standard for drinking water in China (GB 5749-2006) considers Ag^+^ levels below 50 μg L^–1^ to be safe. Each AgNPs treatment was performed with 5 repeats.

### Statistical analysis

The experiments were performed using a completely randomized design (Fernandez et al., 2022). Statistical analyses were carried out using SPSS Statistics 21 (SPSS Inc., United States), and results were showed as mean ± standard error (SE). Student’s *t*-test or one-way ANOVA with Duncan’s multiple range test (*P* < 0.05) were used to determine the significant difference (Zhuo et al., 2022).

## Results

### Antifungal effect of silver nanoparticles against four pathogens on mycelial growth

**FIGURE 1:**
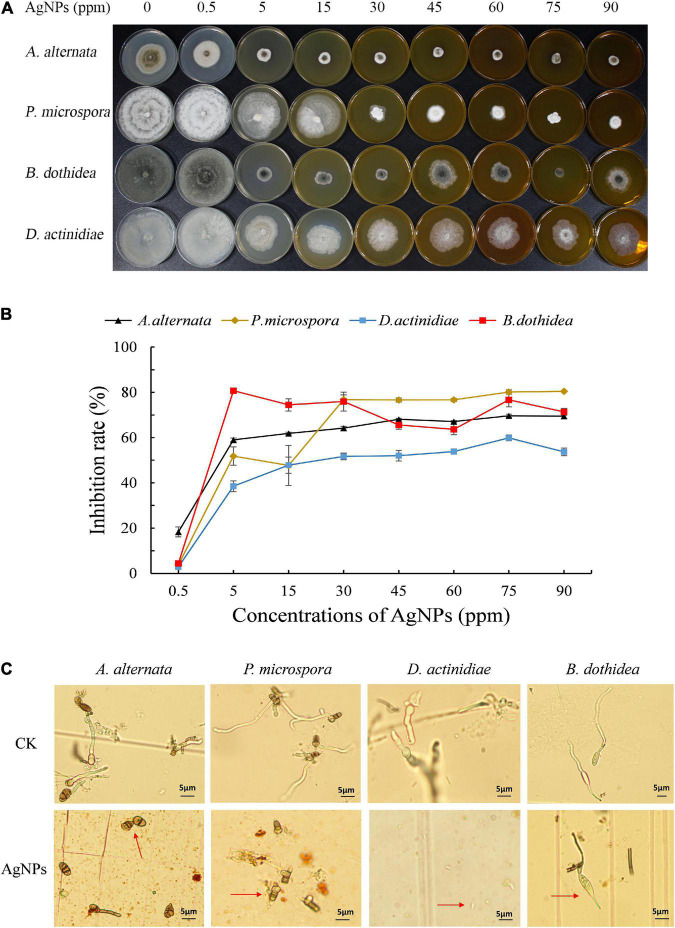
**(A)** The colony reduction and **(B)** Inhibition rate of four pathogens in PDA medium with various AgNPs concentrations. **(C)** Effect of 75 ppm AgNPs on four pathogens’ spore germination and germ tube elongation.

Different concentrations of AgNPs (5 nm, 5 mg mL^–1^) had distinct inhibitory effects on the mycelial growth of four pathogens (Figures 1A,B). Under 60–90 ppm AgNPs, an obvious reduction of colony size was observed, and the inhibition rate of 75 ppm AgNPs achieved a stable and nearly the highest value for four pathogens. Hence, we chose 75 ppm AgNPs for further analysis. Additionally, EC_50_ values for mycelial growth of AgNPs for the four pathogens ranged from 2.31 to 14.74 ppm (Supplementary Table 2), suggesting that AgNPs possessed excellent antifungal activity.

### Effect of silver nanoparticles against pathogens on spore germination and germ tube

The germination assay also revealed that 75 ppm AgNPs significantly reduced the spore germination of four pathogens (Figure 1C). The average spore germination rate of *A. alternata* decreased from 88.48 to 9.70%, and the length of the germ tube decreased from 36.10 to 18.69 μm. Also, the average spore germination of *B. dothidea* decreased from 94.44 to 7.07%, and the length of the germ tube decreased from 66.00 to 17.91 μm. The spore germination of *P. microspora* and *D. actinidiae* were completely inhibited.

### Effect of silver nanoparticles against pathogens on membrane permeability of mycelium

**FIGURE 2:**
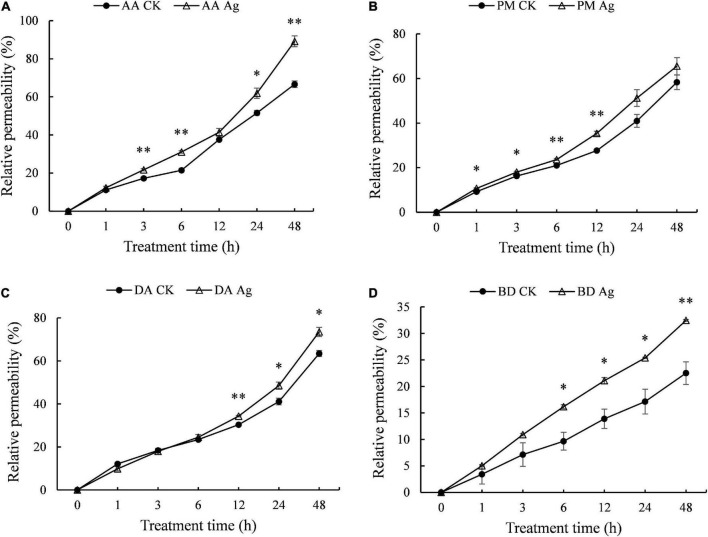
Effects of 75 ppm AgNPs on mycelium membrane permeability of four pathogens’ mycelium. **(A)** *A. alternata*, **(B)** *P. microspora*, **(C)** *D. actinidiae*, **(D)** *B. dothidea. Asterisk*s indicate significant difference (*P* < 0.05*, *P* < 0.01^**^) between the treatments determined using Student’s *t*-test.

Electrical conductivity is used to measure ion strength in solution, which reflects electrolyte exchange inside and outside mycelium cells, as well as the permeability of mycelium cell membrane, which is positively related to the integrity of cell membrane. Compared to the control, the electrical conductivity of four pathogens’ mycelium increased significantly with AgNPs treatment time extension (Figure 2 and Supplementary Table 3), indicating increased permeability of cell membrane and leakage of intracellular substance.

### Effect of silver nanoparticles on the mycelia morphology and ultrastructure of pathogens

**FIGURE 3:**
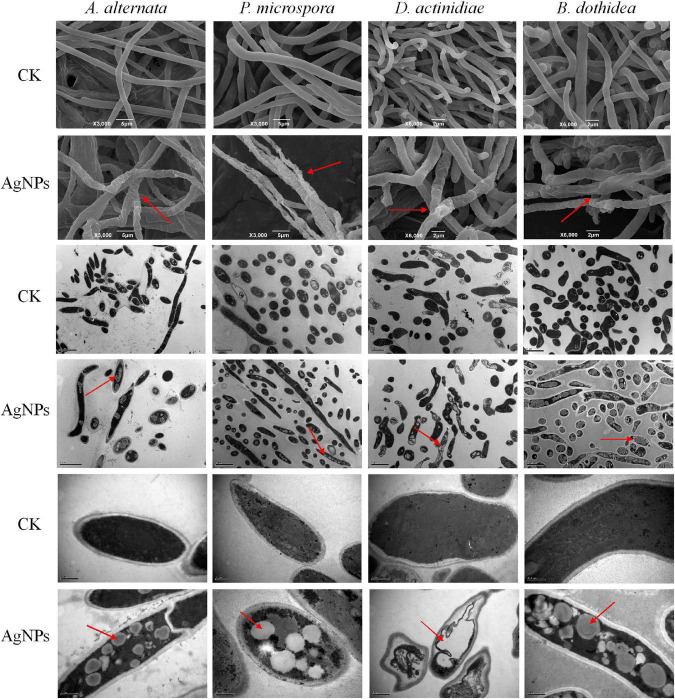
Scanning and transmission electron microscopy images of four pathogens’ mycelial morphology and ultrastructure under 75 ppm AgNPs.

The microstructure changes of four pathogens were examined using SEM and TEM, striking changes in mycelia morphology and cell structure were observed after AgNPs treatment. Under SEM observation (Figure 3), untreated mycelia possessed normal morphology with uniform thickness and smooth surface. In comparison to the control, AgNPs caused abnormal morphological changes in the hypha, which became twisted, shrunken, and shriveled. The abnormal mycelial morphology was linked to a change in the permeability of the mycelium cell membrane. Under TEM observation, the untreated cell structure was unaltered, with a smooth cell wall and few intracellular cavities. AgNPs caused significant cellular damage, including obvious vacuolation and structure loosening. Overall, AgNPs treatment could have a significant detrimental influence on the morphology and ultrastructure of four pathogens.

### RNA-seq analysis

**FIGURE 4:**
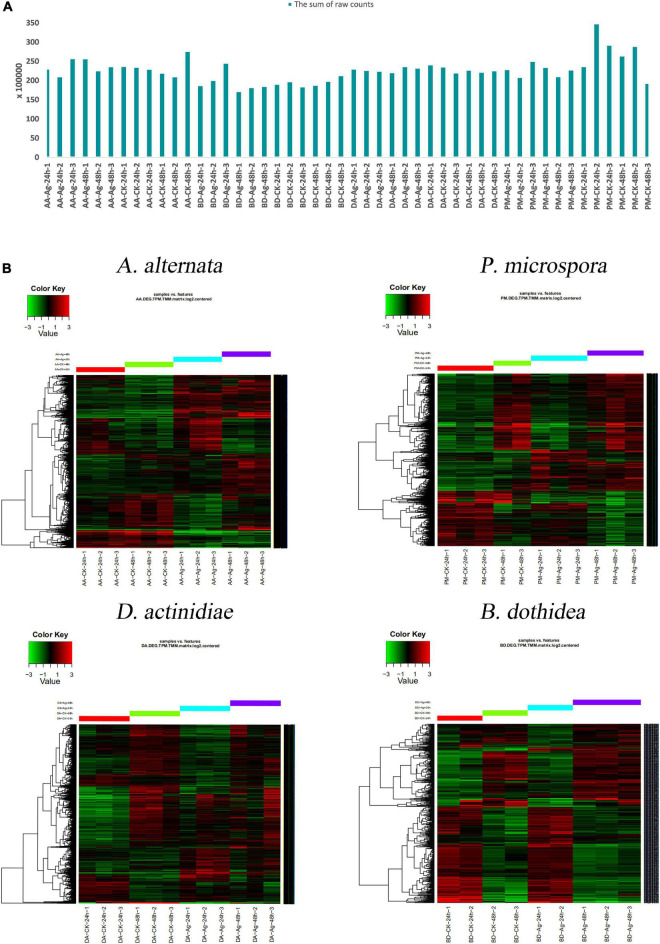
**(A)** The sum of 48 samples’ raw counts. **(B)** Hierarchical clustering analysis of DEGs with different AgNPs exposure time.

Transcriptome profiling assays were performed to uncover the molecular changes underlying the impact of AgNPs on four pathogens. The average raw data size of samples was 9.67 G, the percentage of clean data was 97.67%, and the average mapping rate with the corresponding reference genome reached 95.95% (Figure 4A and Supplementary Table 4). When compared to the genome, the gene number of *A. alternata* was 13466, *P. microspora* was 14711, *D. actinidiae* was 17176, and *B. dothidea* was 14017. TPM and TMM analysis were used to determine the amount of gene expression, and the sum of raw counts for each sample was listed in Supplementary Tables 5–9.

### Significant differentially expressed genes analysis

**FIGURE 5:**
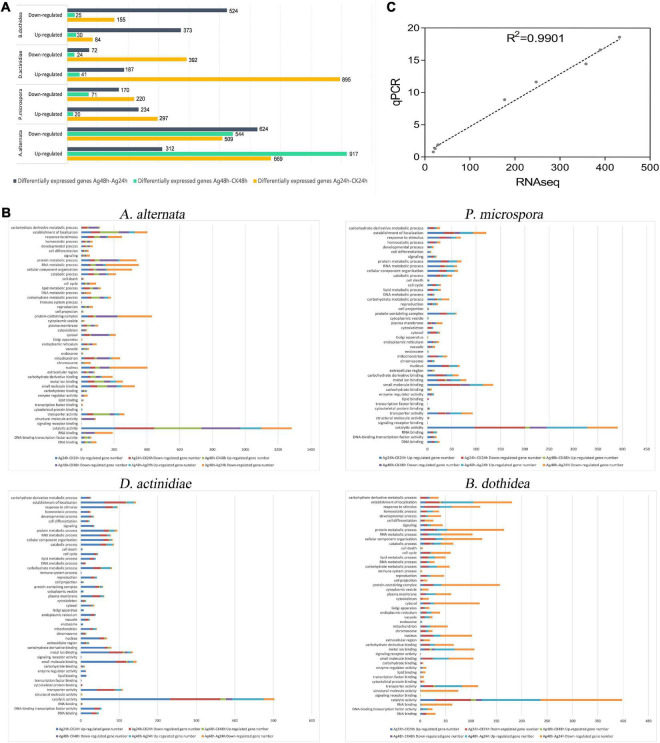
**(A)** Gene expression differences in the AgNPs treated groups compared with the control group. **(B)** GO enrichment analysis of DEGs between Ag-24h and CK-24h. **(C)** Correlation of expression levels of 8 DEGs assessed by RT-qPCR with RNA-Seq data.

Each pathogen’s four treatments were subjected to differential expression analysis (Figure 5A). As shown below, the number of DEGs was determined using cluster analysis (Supplementary Table 10).

Compared to the untreated group (CK), the transcriptome data suggested that (i) For *A. alternata*, 669 and 917 genes were up-regulated when comparing Ag-24h to CK-24h and Ag-48h to CK-48h, respectively; 312 genes were up-regulated when comparing Ag-48h to Ag-24h. (ii) *For P. microspora*, 297 and 20 genes were up-regulated when comparing Ag-24h to CK-24h and Ag-48h to CK-48h, respectively; 234 genes were up-regulated when comparing Ag-48h to Ag-24h. (iii) For *D. actinidiae*, 895 and 41 genes were up-regulated when Ag-24h was compared to CK-24h and Ag-48h was compared to CK-48h, respectively; 187 genes were up-regulated when Ag-48h was compared to Ag-24h. (iv) For *B. dothidea*, 84 and 30 genes were up-regulated when comparing Ag-24h to CK-24h and Ag-48h to CK-48h, respectively; 373 genes were up-regulated when comparing Ag-48h to Ag-24h.

In addition, the transcriptome data analysis revealed that (i) for *A. alternata*, 509 and 544 genes were down-regulated when Ag-24h was compared to CK-24h and Ag-48h was compared to CK-48h, respectively; 624 genes were down-regulated when Ag-48h was compared to Ag-24h. (ii) For *P. microspora*, 220 and 71 genes were down-regulated when comparing Ag-24h to CK-24h and Ag-48h to CK-48h, respectively; 170 genes were down-regulated when comparing Ag-48h to Ag-24h. (iii) For *D. actinidiae*, 392 and 24 genes were down-regulated when comparing Ag-24h to CK-24h and Ag-48h to CK-48h, respectively; 72 genes were down-regulated when comparing Ag-48h to Ag-24h. (iv) For *B. dothidea*, 155 and 25 genes were down-regulated when comparing Ag-24h to CK-24h, and 524 genes were down-regulated when comparing Ag-48h to Ag-24h.

### Cluster analysis of differentially expressed genes

Using hierarchical cluster analysis, the relationship of genome-wide expression profiles between control and AgNPs treated samples of 24 h and 48 h was determined. For each pathogen, all annotated DEGs from the 48 samples were clustered with the software Cluster 3.0 (Figure 4B). The results revealed that the DEG data of different treatments (CK-24h, CK-48h, Ag-24h, Ag-48h) exhibited a similar pattern, the repetition of different treatments were gathered together, indicating that the data was highly reliable.

With the exception of *A. alternata*, the DEGs number of Ag-24h samples was more than that of Ag-48h samples in *D. actinidiae* and *P. microspora*. Fewer DEGs were identified both in Ag-24h and Ag-48h samples of *B. dothidea.* The result indicated that the effective time for AgNPs treatment concentrated within 24 h. Furthermore, when up-regulated and down-regulated genes from the two time periods were combined, only a few genes increased or decreased continuously as the AgNPs treatment time was prolonged. It was assumed that AgNPs treatment had a significant impact on various DEGs within 24 h (Supplementary Figure 1).

### Gene Ontology functional annotation and Kyoto Encyclopedia of Genes and Genomes enrichment analysis of metabolic pathways

The RNA-seq unigenes from all groups were annotated using the NR, Swiss-Prot, KOG, eggNOG, InterPro, Pfam, GO, and KEGG databases (Supplementary Table 11). The responses of four pathogens to AgNPs were analyzed using GO functional enrichment (Figure 5B). Based on GO analysis, the unigenes of four pathogens were classified into three major categories: molecular function, cellular components, and biological process, with 16, 16, and 21 GO functional subcategories, respectively (Supplementary Tables 12–15). The majority of DEGs in the four pathogens were involved in “catalytic activity,” “small molecule binding,” “metal ion binding,” “transporter activity,” “cellular component organization,” “protein metabolic process,” “carbohydrate metabolic process,” and “establishment of localization.”

Kyoto Encyclopedia of Genes and Genomes analysis was used to discover the biological pathways in which the unigenes are involved. For four pathogens, the most highly enriched pathways for these DEGs were “carbohydrate metabolism,” “amino acid metabolism,” “energy metabolism,” “xenobiotics biodegradation and metabolism” of “metabolism processes.” “Cellular processes” were especially enriched in *B. dothidea* (Supplementary Tables 16–19).

The up-regulated DEGs of Ag24h-CK24h in *A. alternata* were mostly involved in “enzyme activity processes,” such as “NADPH,” “cutinase,” and “endo-1,4-beta-xylanase.” The up-regulated DEGs of Ag48h-Ag24h treatment were found in “transcription,” “folding, sorting, and degradation” of “genetic information processing,” respectively. The majority of Ag48h-CK48h DEGs, as well as up-regulated Ag24h-CK24h and Ag48h-Ag24h DEGs, were primarily involved in the “metabolism process,” which included “energy metabolism,” “carbohydrate metabolis,” and “amino acid metabolism.” Partial DEGs were identified in the fields of “environmental information processing,” and “organismal systems” (Supplementary Table 16).

The up-regulated DEGs of Ag48h-CK48h and down-regulated DEGs of Ag48h-Ag24h cannot enrich in KEGG analysis of *P. microspora.* The up-regulated DEGs of Ag24h-CK24h were identified in the “tyrosinase activity process.” The Ag24h-CK24h down-regulated DEGs were mainly involved in “amino acid metabolism,” “xenobiotics biodegradation and metabolism” of “metabolism processes.” The Ag48h-CK48h down-regulated DEGs were identified in the “transport and catabolism” of “cellular processes.” Furthermore, the majority of up-regulated Ag48h-Ag24h DEGs were involved in “metabolism processes,” such as “glycan biosynthesis and metabolism,” “energy metabolism” and “carbohydrate metabolism.” Partial DEGs were identified in the “transport and catabolism” of “cellular processes,” and “membrane transport” of “environmental information processing” (Supplementary Table 17).

The DEGs of Ag48h-CK48h and the up-regulated DEGs of Ag48h-Ag24h cannot enrich in KEGG analysis for *D. actinidiae*. The Ag24h-CK24h up-regulated DEGs were identified in the “enzyme activity processes,” such as “choline dehydrogenase” (EC:1.1.99.1), “mannan endo-1,6-alpha-mannosidase” (EC:3.2.1.101), “prostaglandin reductase 3” (EC:1.3.1.48), and “NADPH-cytochrome P450 reductase” (EC:1.14.14.1). Down-regulated DEGs in Ag24h-CK24h and Ag48h-Ag24h were mainly involved in the “metabolism processes,” including “carbohydrate metabolism,” “amino acid metabolism,” “lipid metabolism,” and “xenobiotics biodegradation and metabolism” (Supplementary Table 18).

The DEGs of Ag48h-CK48h cannot enrich in KEGG analysis for *B. dothidea*. Ag24h-CK24h up-regulated DEGs were found in “transport and catabolism” of “cellular processes,” whereas Ag24h-CK24h down-regulated DEGs were identified in “cell growth and death” of “cellular processes,” and “carbohydrate metabolism” of “metabolism processes.” The Ag48h-Ag24h up-regulated DEGs were mainly involved in “cell growth and death” and “cellular community-eukaryotes” of “cellular processes.” Down-regulated Ag48h-Ag24h DEGs were identified in “biosynthesis process of other secondary metabolites” and “amino acid metabolism” of “metabolism processes” (Supplementary Table 19).

### Validation of RNA-seq

The correlation between qRT-PCR expression levels of eight DEGs and RNA-Seq data was 0.9438, indicating that qRT-PCR results were consistent with RNA-Seq data (Figure 5C and Supplementary Table 20).

### Disease control effect of silver nanoparticles on kiwifruit

**FIGURE 6:**
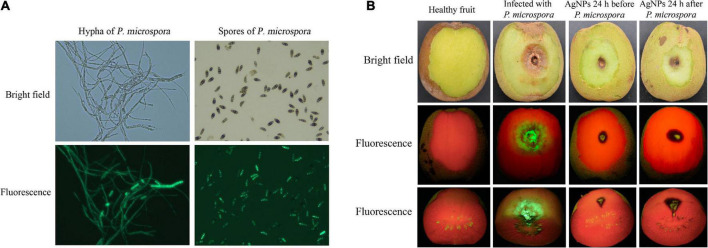
**(A)** Hypha and spores of the wild and fluorescent *P. micropora* strain. **(B)** Antifungal activity of AgNPs on kiwifruit soft rot caused by fluorescent *P. micropora*.

The fluorescence strain PM-13 has no significant differences in morphological characteristics and pathogenicity when compared to wild-type *P. microspora* strain, and the fluorescence intensity was positively related to spore and hyphae colonization, making it more suitable for observing pathogen infection processes, as well as the antifungal activity of AgNPs on controlling kiwifruit post-harvest rot (Figure 6A).

As illustrated in Figure 6B, the average lesion diameter of infected spot without AgNPs treatment was 24.22mm. The average lesion diameter that spraying AgNPs before PM-13 inoculation was 6.23 mm, whereas the average lesion diameter that spraying AgNPs after PM-13 inoculation was 8.90 mm. As a result, spraying AgNPs prior to spore inoculation had a better disease prevention effect (74.29% reduction in lesion diameter) than spraying AgNPs after inoculation (63.26% reduction in lesion diameter). Furthmore, spraying AgNPs reduced not only the average lesion of infected spots on the surface, but also the infected depth of disease spots, as observed in the cross-section. The findings revealed that AgNPs treatment greatly reduced the infected phenotype and fluorescence symptoms, as well as the pathogen colonization and disease development of kiwifruit post-harvest rot (Supplementary Table 21).

There was few Ag^+^ residue in the fruit’s peel or inner/external pulp. The Ag^+^ content in the AgNPs treated samples ranged from 0.033 to 1.499 μg L^–1^, which was significantly lower than China’s national hygienic limit for drinking water (50 μg L^–1^), indicating that AgNPs treatment was safe for kiwifruit (Supplementary Table 22).

## Discussion

Post-harvest rot is one of the most serious fungal diseases encountered during kiwifruit storage, resulting in massive economic losses. Chemical control is currently the most prevalent method of control, despite being hazardous to humans and the environment. In recent years, AgNPs have gradually been applied in the control of agricultural diseases due to their high efficiency and broad-spectrum antimicrobial activities, low resistance, and high safety (Sharma et al., 2018; Malandrakis et al., 2019; Chen et al., 2020). This report firstly indicated the antifungal activity of AgNPs against four pathogens causing kiwifruit post-harvest rot from physiological, cytological, and transcriptomic levels. AgNPs significantly inhibited mycelial growth and spore germination of four pathogens, increased the permeability of mycelium’s cell membrane, caused cellular and organelle structural degradation. Transcriptomic analysis revealed that AgNPs influenced the carbohydrate metabolism, TCA cycle and energy metabolism pathways. In addition, AgNPs remarkably reduced the symptoms of kiwifruit rot without leaving any Ag^+^ residue on the peel and flesh. These findings implied that AgNPs might be a potential substitute for post-harvest disease control.

Silver nanoparticles inhibit both mycelial growth and spore germination of several plant pathogenic fungi, including *B. cinerea, Bipolaris sorokiniana, Magnaporthe grise, Colletotrichum gloeosporioides, F. culmarum, Alternaria* sp., *Cladosporium* sp., *Corynespora* sp., *Cylindrocarpon* sp., *Stemphylium* sp. and *Pythium* sp. (Ganash et al., 2018; Malandrakis et al., 2019; Kasprowicz et al., 2020). Based on the colony diameter and inhibition curve, the inhibitory effect of AgNPs on four pathogens was positively correlated with AgNPs concentration, and the inhibitory effects of AgNPs on four pathogens were *P. microspora* > *B. dothidea* > *A. alternata* > *D. actinidiae.* The findings were consistent with our previous research, as *D. actinidiae* had been identified as the most virulent strain among the four pathogens (Li et al., 2017c). The EC_50_ of AgNPs on the four pathogens was less than 15 ppm, as well as the spore germination and germ tube length were significantly reduced under 75 ppm, showing AgNPs’ strong inhibitory effect on kiwifruit post-harvest rot.

Previous cytological research confirmed that AgNPs severely damaged the cellular components of cell wall of pathogenic fungi, degraded the structure and integrity of cell membrane, and caused spores and mycelia to become wrinkled and depressed. Then, oxygen consumption of mycelial respiration reduced, the inactivation of key enzymes affected the respiratory chain, and bound lipids and enzymes caused cell lysis. Eventually, the cells twisted and expanded until they withered and died (Xu et al., 2013; Dakal et al., 2016; Xia et al., 2016; Gordienko et al., 2019). The electrical conductivity analysis in our study revealed that AgNPs treatment increased cell membrane permeability, destroyed the integrity of the cell membrane of four pathogens, resulting in excess water entering the cells and introducing the expansion of cell volume due to osmotic pressure, ultimately leading to the exudation of the cellular contents. SEM and TEM analysis further confirmed that AgNPs treatment caused hypha to become twisted, shrunken, and shriveled. Additionally, treated cells became loose, and vacuolations occurred. Consistent with morphological observations, the results showed that AgNPs severely damaged the cellular and organelle structure of four pathogens.

Over the past 2 years, transcriptomic analysis has gradually been used to clarify the molecular mechanism of AgNPs, providing a comprehensive understanding of potential DEGs involved in specific biological processes (Lu et al., 2020; Rahman et al., 2022). For example, the molecular mechanism of AgNPs against *Fusarium* sp. has been deeply studied, AgNPs could impair the cell structure, cellular energy utilization, energy and substance metabolism, signal transduction pathways, and significantly induce the expression of azole-related ATP-binding cassette (ABC) transporters (Shen et al., 2020; Jian et al., 2021). In our study, AgNPs caused 1178 and 1461 DEGs in *A.alternata*, 517 and 91 DEGs in *P. microspora*, 1287 and 65 DEGs in *D. actinidiae*, and 239 and 55 DEGs in *B. dothidea* at 24 h and 48 h, respectively. For four pathogens, there was a common gene expression of KEGG categories associated with “carbohydrate metabolism,” “amino acid metabolism,” “energy metabolism,” and “xenobiotics biodegradation and metabolism” of “metabolism processes.” Besides that, *B. dothidea* was particularly enriched in “cellular processes.” The findings demonstrated that AgNPs altered the pathogens’ gene expression, most of which were involved in, the foundation of all living organisms’ activities.

Carbohydrates are essential components of cell membranes and skeletons, as well as an energy source (Diao et al., 2020). The cell wall and membranes, which were composed of extensively cross-linked glycoproteins and carbohydrates, had been identified as the primary targets of AgNPs (Gow et al., 2017; Barros et al., 2021). Our findings indicated that nearly all DEGs encoding “carbohydrate metabolism” were repressed in the AgNPs-exposed group of four pathogens, which was consistent with the mechanism of AgNPs against *Articulospora tetracladia* (Barros et al., 2021) and *F. solani* (Shen et al., 2020). For *A. alternata*, the DEGs encoding “ascorbate and aldarate metabolism,” “galactose metabolism,” “glyoxylate and dicarboxylate metabolism,” “pyruvate metabolism,” “glycolysis and gluconeogenesis,” and “pentose phosphate pathway” were down-regulated in all AgNPs-exposed groups. DEGs encoding “fructose and mannose metabolism,” “glyoxylate and dicarboxylate metabolism,” and “pentose and glucuronate interconversions” in *D. actinidiae* were down-regulated in the Ag24h-CK24h group. The DEGs encoding “amino sugar and nucleotide sugar metabolism” and “starch and sucrose metabolism” in *B. dothidea* were down-regulated in the Ag24h-CK24h and Ag48h-Ag24h groups. Combined with our physiological and microscopic results, we can further confirm that AgNPs significantly disrupted the “carbohydrate metabolism” of four pathogens, and destroyed the cell membrane integrity of four pathogens.

Silver nanoparticles treatment also disrupted the “amino acid metabolism” and “lipid metabolism” of four pathogens. Except for *B. dothidea*, the DEGs involved in “amino acid metabolism” in the other three pathogens were down-regulated, including “cysteine and methionine metabolism,” “lysine metabolism,” “arginine and proline metabolism,” “valine, leucine and isoleucine biosynthesis,” “alanine, aspartate and glutamate metabolism.” The change of amino acid-related metabolism pathways resulted in protein synthesis disruptions, such as “beta-alanine metabolism” and “arginine andtabolism.” Furthermore, in Ag24h-CK24h and Ag48h-Ag24h of *D. actinidiae*, DEGs encoding “sphingolipid metabolism,” “fatty acid degradation,” “glycerolipid metabolism,” and “lipid metabolism” were down-regulated. The citrate cycle (TCA cycle) is the final metabolic pathway, the link between the metabolic connection of carbohydrates, amino acids, and lipids, as well as the source of energy for life activities (Fernie et al., 2004). Since the TCA cycle of *A. alternata* was significantly down-regulated by AgNPs, we hypothesized that AgNPs induced carbohydrate, amino acid, and lipid degradation, thus influencing the TCA cycle and energy metabolism of four pathogens.

In photosynthesis, the carbon fixation pathway of plants (CFPP) converts solar energy into biomass, bio-products, and biofuel. Surprisingly, a large number of heterotrophic fungi have enzymes that are functionally related to CFPP (Nelson and Ben-Shem, 2004). For example, DEGs encoding “carbon fixation in photosynthetic organisms,” “oxidative phosphorylation,” and “methane metabolism” in *A. alternata* were down-regulated, indicating that the pathway that absorbed and transmited light energy to the photosynthetic reaction center could be an important target of AgNPs. Besides that, oxidative phosphorylation, the cornerstone of aerobic cellular metabolism, was also significantly suppressed in *A. alternata*. These findings indicated that AgNPs significantly inhibited the energy metabolism pathways of four pathogens.

The findings also found that four pathogens attempted to eliminate the alterations caused by AgNPs and utilized multiple cellular responses to AgNPs stress. For example, xenobiotics detoxification systems can remove compounds from the complex mixtures formed by metabolic processes or from the environment. Cytochrome P450 is involved in more than 90% of all these oxidation reactions and is responsible for the metabolism of a wide range of xenobiotics and endogenous compounds (Zhu et al., 2018; Zhuo and Fan, 2021). In our study, the enzymes involved in “xenobiotics biodegradation and metabolism” were down-regulated in Ag24h-CK24h of *A. alternata*, such as “benzoate degradation,” “caprolactam degradation,” “naphthalene degradation,” “dioxin degradation,” and “polycyclic aromatic hydrocarbon degradation.” Then, as predicted, the metabolism of “xenobiotics by cytochrome P450” was up-regulated after 24 h, similarly found in *Phanerochaete chrysosporium* under AgNPs stress (Liu et al., 2022). In addition, there was partial recovery of “carbohydrate metabolism,” “amino acid metabolism,” lipid metabolism” and “energy metabolism” after 24 h, which might be related to the defense mechanism of four pathogens against AgNPs, similar to that of *Fusarium solani* (Shen et al., 2020).

Differ from the other pathogens, the “cellular processes” of *B. dothidea* were particularly enriched. Previous research found that AgNPs caused massive cell cycle arrest, a high percentage of DNA breaks, and cell death (Quevedo et al., 2021). The DEGs of “cellular senescence,” “cell growth and death,” “cignaling pathways regulating pluripotency of stem cells,” and “cellular community-eukaryotes” were up-regulated after 24 h. In subsequent research, we will increase the treatment concentration and sampling time point of AgNPs on each pathogen, investigate the antifungal mechanism of AgNPs against each pathogen, and confirm the function and mechanism of the critical DEGs involved in morphological and cytological changes of four pathogens.

Many field experiments confirmed that the spraying time of AgNPs affected the inhibition effect. Spraying 10 ppm AgNPs on rose 2 days before pathogen inoculation reduced the incidence rate of powdery mildew by 95% (Kim et al., 2008); spraying AgNPs 3 h before pathogen inoculation significantly reduced the incidence of root rot and rice blast (Jo et al., 2009). Our findings confirmed that AgNPs significantly reduced the severity of kiwifruit post-harvest rot, as the lesion diameter of disease spot and fluorescence intensity of colonized hyphae treated with AgNPs were obviously smaller than those of the control. Consistent with previous studies, spraying AgNPs on fruit prior to pathogen infection had a significantly better control effect than spraying AgNPs after pathogen infection. Meanwhile, there was no Ag^+^ residue on the peel or flesh of kiwifruit, indicating that AgNPs could be safely applied during the fruiting period in the field and the fruit preservation period after harvest.

Antifungal activity of AgNPs is dependent on physical and chemical properties, such as particle size, shape, dispersion, and pH (Hong et al., 2016). It was confirmed that AgNPs with a diameter of less than 12 nm could directly penetrate the cell wall and enter the cytoplasm of pathogens (Raza et al., 2017; Noori et al., 2017). AgNPs with an average size of 4–8 nm with various concentrations (10–100 ppm) significantly inhibited *Colletotrichum* sp. and *Alternaria* sp. (Lamsal et al., 2011; Gupta and Chauhan, 2015). Furthermore, spherical AgNPs could effectively inhibit fungi growth at low concentrations (Kim et al., 2012; Akther and Hemalatha, 2019). AgNPs in our study was 5 nm in size and spherical, demonstrating that they can be effectively used to control kiwifruit post-harvest rot. This finding can be used to guide the selection and application of AgNPs for horticultural disease control.

## Conclusion

In summary, our results aimed at reliving the threat of serious fungal post-harvest rot in the kiwifruit industry, we performed an in-deep analysis of the antifungal effect of AgNPs against four pathogens from physiological, cytological, and transcriptomic levels, and verified that AgNPs could be effectively used to control kiwifruit post-harvest rot and safely applied on kiwifruit without residue. According to the findings, AgNPs are potentially suitable for use in the development of new antifungal agents for combating plant fungal diseases, including horticultural crops that directly consume fruits.

## Data availability statement

The datasets presented in this study can be found in online repositories. The names of the repository/repositories and accession number(s) can be found below: https://ngdc.cncb.ac.cn/search/?dbId=gsa&q=CRA007558.

## Author contributions

LL: methodology, investigation, software, writing – original draft, and funding acquisition. HP: methodology, investigation, software, and editing. LD: data curation and formal analysis. GQ: formal analysis, supervision, and editing. ZW: data curation and software. WL: methodology. CZ: project administration, supervision, editing, and funding acquisition. All authors contributed to the article and approved the submitted version.

## Footnotes

1
http://geneontology.org/

2
https://www.genome.jp/kegg/
