# Supplementary material for: The antifungal activity and mechanism of silver nanoparticles against four pathogens causing kiwifruit post-harvest rot
Source: Front Microbiol. 2022 Aug 31;13:988633. doi: 10.3389/fmicb.2022.988633 (PMC9471003; doi:10.3389/fmicb.2022.988633)
Supplement: Supplementary file 2 [file Data_Sheet_1.docx]

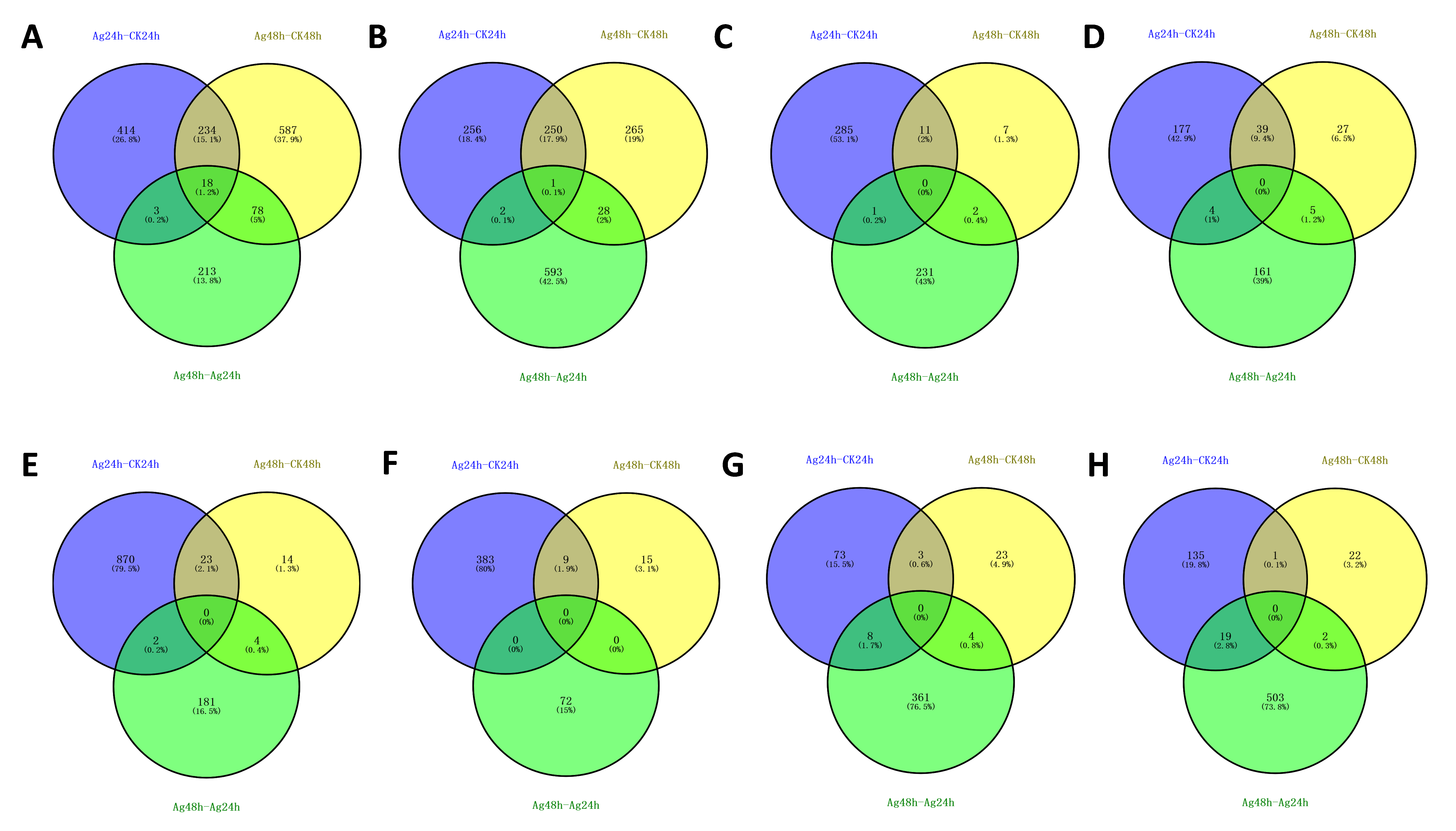


**Supplementary Figure 1** Venn diagram showed the number of DEGs crossed after 24h or 48h treatments. (A/B) Up-regulated/down-regulated genes of *A. alternata*; (C/D) Up-regulated/down-regulated genes of *P. microspora*; (E/F) Up-regulated/

down-regulated genes of *D. actinidiae*; (G/H) Up-regulated/down-regulated genes of *B. dothidea*.
