# Supplementary figures and images for: The antifungal activity and mechanism of silver nanoparticles against four pathogens causing kiwifruit post-harvest rot
Source: Front Microbiol. 2022 Aug 31;13:988633. doi: 10.3389/fmicb.2022.988633 (PMC9471003; doi:10.3389/fmicb.2022.988633)

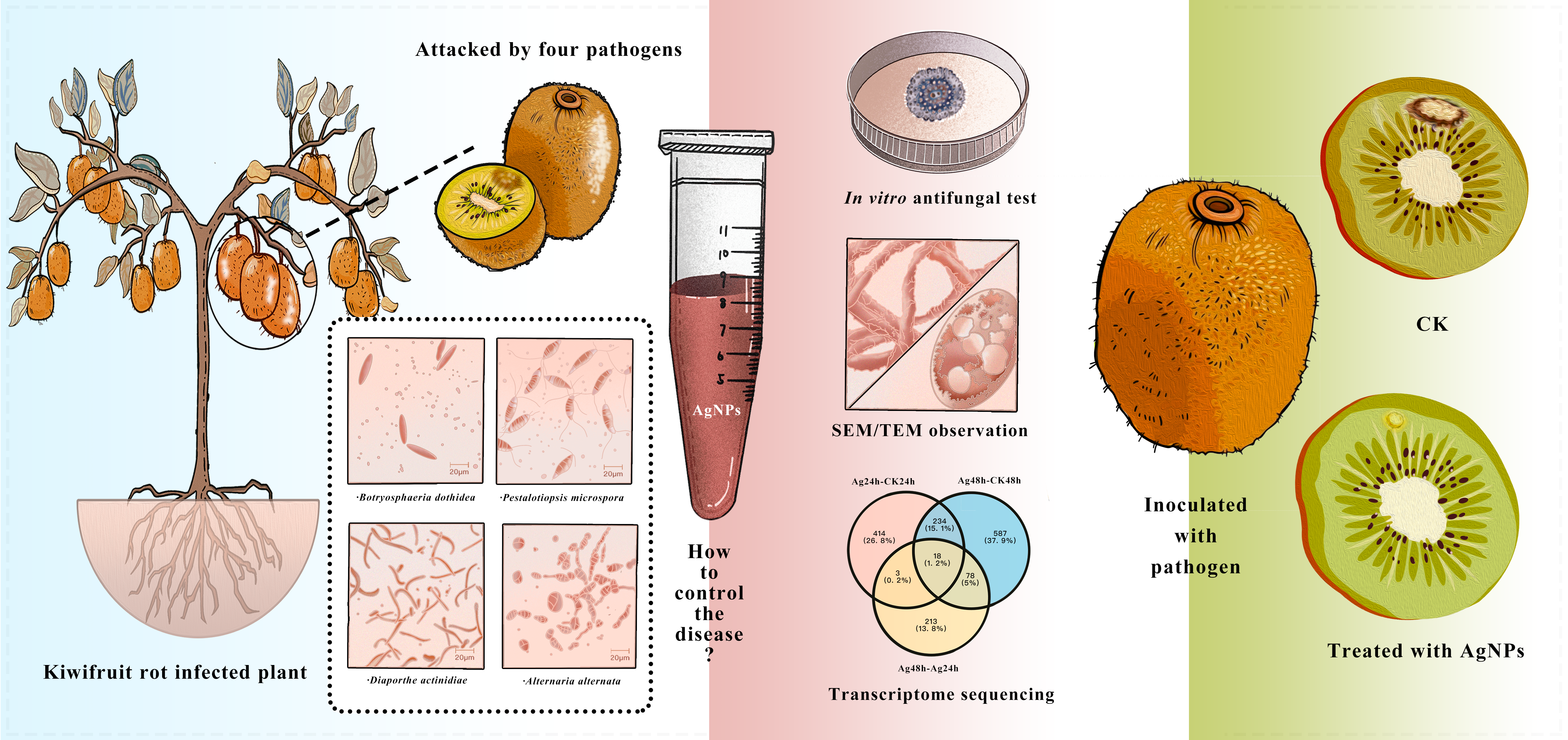

Supplement: Supplementary file 3 [file Image_1.PNG]
